# Physicians’ Resilience as a Positive Effect of COVID-19

**DOI:** 10.31662/jmaj.2022-0192

**Published:** 2023-10-05

**Authors:** Tomoyuki Ushimoto, Kenshi Murasaka, Masaru Sakurai, Masao Ishizaki, Yukihiro Wato, Tsugiyasu Kanda, Yuji Kasamaki

**Affiliations:** 1 Department of Emergency Medicine, Kanazawa Medical University, Uchinada, Japan; 2 Department of Community Medicine, Kanazawa Medical University, Uchinada, Japan; 3 Department of General Medicine, Kanazawa Medical University Himi Municipal Hospital, Himi, Japan

**Keywords:** resilience, physician, COVID-19, post-traumatic growth

## Abstract

People devoid of COVID-19 may exhibit mental health problems, such as anxiety disorders, depression, panic attack, insomnia, emotional disorder, and suicidal actions. Healthcare workers (HCWs) may also exhibit these problems. Physicians should be careful an “at-risk” population. Physicians revealed higher levels of resilience than the popular workers. Humans with stronger resilience have lower feeling of anxiety and depression. We investigated the risk to physicians from an infected environment to infected patients during the pandemic. The social and psychological support of all HCWs, particularly physicians, is significant in the fight against this pandemic. Physicians working with patients with COVID-19 should set enough time to relax, sleep, and spend time with family. Resilience in physicians facing COVID-19 can induce post-traumatic growth in the future.

## Introduction

Psychiatrist Viktor Frankl’s memoir has riveted generations of readers with descriptions of life in Nazi death camps and lessons for spiritual survival ^(1)^. On the basis of his own experience and the stories of his patients, Frankl argues that we cannot avoid suffering, but we can choose how to cope with it, find meaning in it, and move forward with a renewed purpose.

In this study, we investigate how people cope during the COVID-19 pandemic, even with physicians facing infected patients. We investigated the risk to physicians from an infected environment to infected patients during the pandemic. In particular, the study reviewed the resilience points of physicians holding mental disturbances in their lives, even during the COVID-19 pandemic. We expect that resilience in physicians facing COVID-19 can induce post-traumatic growth (PTG) in the future.

PTG can be after situations such as life threat. Instead of a general moral injury, healthcare workers (HCWs) may be faced with a level of moral distress. Moral distress induces post-traumatic stress, which is necessary for PTG to occur. In this review, we discuss the relation among resilience, PTG, and post-traumatic stress.

## COVID-19 Pandemic Crisis

People affected by COVID-19 may have a high burden of mental health problems, such as depression, anxiety disorders, stress, panic attack, irrational anger, impulsivity, somatization disorder, sleep disorders, emotional disturbance, post-traumatic stress symptoms, and suicidal behavior. HCWs may also exhibit these problems ^(2)^. Lai et al. have reported a 50.4% depression rate among those working during the COVID-19 pandemic. Several studies have also explored the mental health problems of HCWs during the COVID-19 pandemic ^(3)^. The COVID-19 pandemic has caused additional psychological difficulties and increased the workload of HCWs. HCWs face unprecedented rates of COVID-19-related psychological stress in both professional and personal domains.

In Japan, research on the mental health of patients with COVID-19 found that neuroticism is a positive predictor of stress and anxiety ^(4)^. HCWs in Japanese national university hospitals were surveyed to determine whether a participant engaged in the care of patients with COVID-19 in the past 2 weeks. Of those who engaged in the care of patients with COVID-19, 50% reported burnout, whereas 6.1% did not ^(5)^. In a cross-sectional study, Matsuo et al. investigated the prevalence of burnout among HCWs in Japan during COVID-19, which reported an overall prevalence of burnout of 31.4% ^(6)^.

Medicine is one of the occupations that lead to higher levels of anxiety, depression, suicide, stress, and divorce. Physicians may be considered an “at-risk” population, with higher rates of depression, anxiety, suicide, divorce, stress, and emotional exhaustion than other segments of the population ^(7)^. The pandemic also had a negative impact on medical students and newly graduated physicians. Exposure to COVID-19 and isolation from family induces anxiety symptoms.

In graduate physicians, alcohol intake increased ^(8)^. Although the national survey study suggested that physicians exhibited higher levels of resilience than the general working population, the recent review showed the prevalence of depression and anxiety was 20.5% and 25.8%, respectively, in medical doctors ^(9), (10)^. The psychological well-being of physicians is of vital significance, not only for physicians themselves but also for the quality of health care they provide.

Although relatively large animal and human studies have shown that chronic exposure of adults to high levels of stress is usually associated with increased susceptibility to mood, anxiety, and addiction disorders, stress affects most behavioral domains with an inverted U-shaped curve. The inverted U-shaped relationship is postulated in the relationship between stress and coping, whereby both low and high levels of stress impair behavior. In contrast, intermediate levels promote positive coping responses in all organisms ^(11)^. Therefore, intermittent exposure to stress may evoke stress resilience.

## Role of Resilience in Crisis

**Figure 1.**
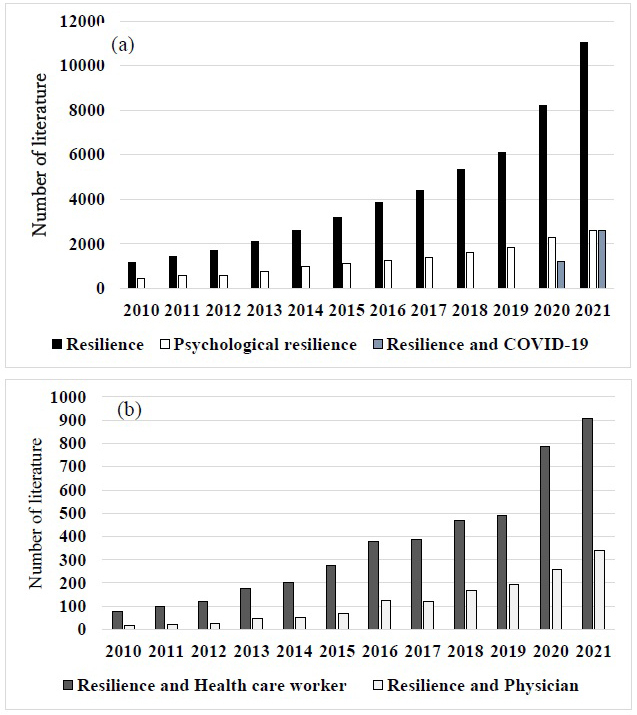
Number of keywords by PubMed in the recent 11 years. (a) The number of keywords, such as resilience, psychological resilience, and COVID-19, is shown. (b) A number of keywords, such as resilience and healthcare workers or resilience and physicians, are shown. Keywords including resilience have increased in recent years.

Figure 1 shows the number of keywords, such as resilience over the 11 years. It is difficult to define resilience. Derived from the Latin word *resilio*―to rebound or bounce back―one definition states that it is the ability to adapt well in the face of adversity or significant stress, even returning stronger afterward. Resilience in doctors was identified as demographics, personality factors, organizational or environmental factors, social support, leisure activities, overcoming previous adversity, and interventions to improve resilience ^(12)^. Resilience protects from workplace stress. When the demands of the workplace exceed the resources of the staff, individuals experience stress. Psychological resilience refers to an individual’s ability to overcome and adapt to adverse conditions when faced with them. Studies have shown that people with greater psychological resilience have lower levels of depression and anxiety ^(13)^. As the psychological resilience levels of physicians decreased, anxiety and depressive symptoms increased ^(14)^.

The psychological and social support of all HCWs, particularly physicians, is important in the struggle with the pandemic. Determining the variables related to psychological resilience in HCWs will be a guide for psychosocial services. Depression and anxiety levels were significantly lower among physicians with higher psychological resilience ^(15)^.

A systematic review on resilience identified five themes of resilience: rising above adversity, adapting and adjusting, resilience as a dynamic process, “ordinary magic,” and mental illness as a marker of resilience ^(12)^. Resilience was initially regarded as an inherited, static character trait. However, research has identified it as a dynamic and transient quality ^(16)^. Importantly, resilience education requires a foundation of self-awareness and the ability to self-monitor ^(7)^. Physicians working with patients with COVID-19 should set enough time to relax, sleep, and spend time with family.

Resilient physicians are supported by a nurturing work culture, teamwork, support from the medical community and at home, and self- and organization-oriented interventions ^(17)^. Psychological resilience was significantly higher in physicians who had children, worked for ≥15 years, and had received training about COVID-19. Depression scores were higher among female physicians and those with chronic diseases, whose workload increased after the outbreak, and who had physical contact with COVID-19-positive patients ^(15)^.

## Resilience Strategies for Future Pandemics

Resilient-based intervention is beneficial to avoid burnout in medical students and postgraduate physicians. In some reports, this intervention had detrimental effects on overall resilience, depending on the resilience levels of the trainee and training. The mood of a physician as a leader is highly contagious and creates a ripple effect in medical institutions. New skills are needed to thrive during the COVID-19 pandemic ^(18)^.

Rieckert et al. recommended building and maintaining the resilience of HCWs exposed to COVID-19 by investigating reviews with 73 articles. They showed that resilience would be built and maintained by optimal provision of education and training ^(19)^. Resilience training and interventions can create a feeling of well-being.

Coping strategies, such as problem- and emotion-focused strategies, and resilience are important to achieve an adaptive effect on the mental health of physicians ^(20)^. The psychological resilience that will serve to capture and critically evaluate the quality of review work and generate a synthesis of health outcomes and potential recommendations across fields of study and modes of practice ^(20)^. Approach-oriented coping, such as active and meaning-focused coping and seeking social support, was minimally predictive of subsequent adjustment. Given the unique and ongoing circumstances of COVID-19, specific interventions targeting psychosocial resources and coping identified here may help promote resilience as the pandemic continues to unfold ^(21)^.

Harnessing positive coping strategies such as exercise, modified routines, and social strategies to improve physical and mental health, foster social support, and encourage meaningful daily activities during times of stress and trauma. Education, coping tools, and therapy to help avoid or alleviate the adverse effects on their well-being ^(22)^. Coping skills may help them change their mood. If you have had a bad day at work, playing with your children or watching a funny movie might cheer you up. Alternatively, if you are angry about something someone has said, a healthy coping strategy might help you calm down before you say something you might regret. Below are some examples of healthy emotion-focused coping skills ^(23)^.

Targeting and improving hope, efficacy, resilience, and optimism can be highly effective in improving well-being and positive functioning at work. Using Qualtrics panel data collected from 3860 employees across 15 nations, PsyCap was found to be strongly associated with workplace proactivity, proficiency, adaptability, and overall work performance across all 15 nations. These results suggest that efforts to develop PsyCap are effective across national cultures and could be a robust approach for enhancing positive functioning in the global workplace ^(24)^.

Healthcare providers can use various strategies to support resilience and mental well-being among frontline healthcare professionals. These could include work-based interventions, such as changing routines or improving equipment, or psychological support interventions, such as counseling ^(23)^.

Resilience has increased, and the general population has found a surprising ability to adapt. Coping mechanisms, such as active attitudes, planning, acceptance, and reinterpretation of reality, are positively associated with life satisfaction. Other factors that have helped reduce stress are protective measures, psychological counselors, team support, stress monitoring, regular breaks, knowledge of the disease, and things becoming easier. The relaxed doctor, who controls the situation with good emotional control and stress resistance, is also reflected by involvement in the professional role ^(25)^. Evidence-based information can reduce COVID-19 threat activity, but insufficient or excessive levels of threat activity may increase the risk of mental or physical health problems ^(26)^.

The concept of physician resilience has six key themes: tenacity, resources, reflective ability, coping skills, control, and growth (Table 1) ^(15)^. This report mentioned that resilience in physicians is dynamic and must be supported not only by physician-directed interventions but also by organization-directed interventions ^(17)^.

**Table 1.** The Concepts of Physician Resilience as Organizational and Personal Interventions.

Themes	Organizational intervention	Personal intervention
Resource	Duty hour limits	Training for team improvement
Tenacity	Control for patient care time	Mindfulness
Control	Flexibility for working time	Grief counseling
Coping	Reduction of unnecessary bureaucracy	Coping skills training
Reflective ability	Debriefing sessions	Skill training
Growth	Mentoring or coaching programs	Post-traumatic growth

Psychological support systems must be prepared and established, including resilience for physicians in the protection against further pandemics, such as unexpected disasters, climate change, and earth-wide wars.

## PTG in the Adversity of COVID-19

Resilience improves personal growth and the perceived professional benefits. Age and work experience are positively related to resilience. Positive attitudes, such as social networks, peer support, teamwork, self-reliance, problem negotiation, and self-care, can reduce stress and boost resilience ^(27)^. To maintain the resilience of physicians exposed to COVID-19 and further unexpected tasks, optimal provision of proper information and resilience training and interventions to create a feeling of belonging should be prepared ^(28)^. The behavioral and biological stress responses may influence and be influenced by feelings of safety that come about through relationships with others and spiritual and place-based connections ^(28)^.

**Figure 2.**
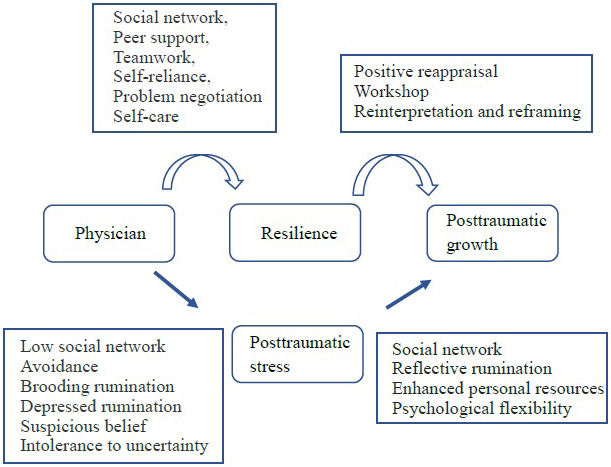
Summary of physician, resilience, and post-traumatic growth.

PTG is expected, even during the COVID-19 pandemic. It differs from resilience in several ways. It is characterized by positive psychological benefits, whereas resilience is characterized by a return to the initial state. Chinese students after the COVID-19 crisis developed PTG, who showed more positive coping and cognitive strategies and maintained higher levels of resilience ^(29)^. During COVID-19, PTG was observed in nurses, medical students, and residents ^(30), (31), (32)^. Subjective well-being was widened by the COVID-19 pandemic in 2020 in Japan through the Online Panel Survey of Social Stratification and Psychology ^(33)^. The coordination of multisectoral support may induce well-being ^(33)^. The higher level of PTG is induced by positive reappraisal, and the workshop induces positive reinterpretation and reframing ^(25)^. The other report showed that coping strategies such as problem-focused, emotion-focused, and avoidance coping mediated the relationship between perceived social support, personality traits, and PTG ^(34)^. PTG often takes a long time to come to fruition. After the COVID-19 pandemic, humans may have undergone epigenetic modifications of resilience through PTG. The mechanism of PTG is based on the subjective exposure of whether or not the event was an experience that shook a person’s values and beliefs. It is important that one’s experience changes perspective on life. The impact is associated with post-traumatic stress induced by several factors, such as poor social network, avoidance, brooding rumination, depressed rumination, suspicious beliefs, and intolerance to uncertainty ^(35), (36), (37), (38)^. After post-traumatic stress, PTG will be built through some factors, such as social network, reflective rumination, enhanced personal resources, and psychological flexibility ^(37), (39), (40)^ (Figure 2). We hope that modified resilience can continue across generations in the event of unexpected disasters.

## Article Information

### Conflicts of Interest

None

### Acknowledgement

We really appreciate the staff in Kanazawa Medical University.

### Author Contributions

KM, MS, and MI organized references and structured this paper. YW and TK reviewed this paper. YK managed and gave the various ideas for making this paper.
